# MiR-455-5p suppresses *PDZK1IP1* to promote the motility of oral squamous cell carcinoma and accelerate clinical cancer invasion by regulating partial epithelial-to-mesenchymal transition

**DOI:** 10.1186/s13046-023-02597-1

**Published:** 2023-02-03

**Authors:** Sheng-Yen Hsiao, Shang-Mei Weng, Jenn-Ren Hsiao, Yi-Ying Wu, Jia-En Wu, Chia-Hao Tung, Wan-Lin Shen, Shu-Fang Sun, Wen-Tsung Huang, Cheng-Yao Lin, Shang-Hung Chen, Tse-Ming Hong, Yuh-Ling Chen, Jang-Yang Chang

**Affiliations:** 1 Institute of Clinical Medicine, College of Medicine, National Cheng Kung University, Tainan, Taiwan; 2 Division of Hematology-Oncology, Department of Internal Medicine, Chi Mei Medical Center, Liouying, Tainan, Taiwan; 3 Department of Otolaryngology, National Cheng Kung University Hospital, College of Medicine, National Cheng Kung University, Tainan, Taiwan; 4 Clinical Medicine Research Center, National Cheng Kung University Hospital, College of Medicine, National Cheng Kung University, Tainan, Taiwan; 5 Department of Pathology, Chi Mei Medical Center, Liouying, Tainan, Taiwan; 6 Department of Senior Welfare and Services, Southern Taiwan University of Science and Technology, Tainan, Taiwan; 7 Department of Environmental and Occupational Health, National Cheng Kung University, Tainan, Taiwan; 8 National Institute of Cancer Research, National Health Research Institutes, Tainan, Taiwan; 9 Department of Oncology, National Cheng Kung University Hospital, College of Medicine, National Cheng Kung University, Tainan, Taiwan; 10 Institute of Oral Medicine, College of Medicine, National Cheng Kung University, Tainan, Taiwan; 11 Institute of Biotechnology and Pharmaceutical Research, National Health Research Institutes, Miaoli, Taiwan; 12 Taipei Cancer Center, Taipei Medical University Hospital, Taipei Medical University, Taipei, Taiwan

**Keywords:** miR-455-5p, PDZK1IP1, Partial epithelial-to-mesenchymal transition

## Abstract

**Background:**

Lymph node and distant metastasis contribute to poor outcomes in patients with oral squamous cell carcinoma (OSCC). The mechanisms regulating cancer migration and invasion play a key role in OSCC.

**Methods:**

We determined migration and invasion ability of OSCC by wound-healing assay, two-chamber transwell invasion assay and cell mobility tracking and evaluated tumor metastasis in vivo. Western blot (WB), qRT-PCR, RNA-seq, dual-luciferase reporter assays and nuclear/cytoplasmic fractionation were performed to investigate the potential mechanism. Immunohistochimical (IHC) staining determined vimentin and *PDZK1IP1* expression in OSCC tissues.

**Results and conclusion:**

In this study, we determined that miR-455-5p was associated with lymph node metastasis and clinical invasion, leading to poor outcomes in patients with OSCC. MiR-455-5p promoted oral cancer cell migration and invasion and induced epithelial-to-mesenchymal transition (EMT). We also identified a new biomarker, *PDZK1IP1* (MAP17), that was targeted by miR-455-5p. *PDZK1IP1* knockdown led to migration, metastasis, EMT, and increased transforming growth factor-β signaling in OSCC. In addition, miR-455-5p overexpression and *PDZK1IP1* inhibition promoted collective OSCC cell migration. According to data from the Cancer Genome Atlas database and the NCKU-OrCA-40TN data set, miR-455-5p and *PDZK1IP1* are positively and negatively correlated, respectively, with partial EMT score. High miR-455-5p expression was associated with high vimentin levels and low MAP17 H-scores. The patients with low MAP17 expression had higher rates of disease recurrence than did patients with high MAP17 expression, especially for patients with clinical invasion risk factors and low MAP17 expression. These results suggest that miR-455-5p suppresses *PDZK1IP1* expression and mediates OSCC progression. MiR-455-5p and *PDZK1IP1* may therefore serve as key biomarkers and be involved in regulating partial EMT in OSCC cells. *PDZK1IP1* expression may also serve as an independent factor that impacts outcomes in patients with clinical risk factors for recurrence.

**Supplementary Information:**

The online version contains supplementary material available at 10.1186/s13046-023-02597-1.

## Background

Oral cancer is a crucial health concern, with its annual incidence exceeding 350,000 [1]. Its prevalence in Taiwan is higher than that in other countries because of its association with smoking, betel nut chewing, and alcohol consumption [2]. Multimodal treatment approaches contributed to more favorable clinical outcomes than ever before. However, patients who experience local/regional recurrence or lymph node or distant metastasis often experience unfavorable clinical outcomes. After a patient’s cancer progresses to metastatic disease, their treatment options are limited. Drugs targeting the Epidermal growth factor receptor signaling pathway, such as cetuximab, have been applied in the treatment of head and neck squamous cell carcinoma (HNSCC) [3]. Immunotherapy is an essential component of cancer therapy, and clinical data have revealed that anti-PD-1 agents, such as nivolumab and pembrolizumab, can improve clinical outcomes in patients with HNSCC [4, 5]. However, few patients have exhibited durable responses to these agents, indicating that such treatments have considerable room for improvement. Therefore, further investigation of different models of carcinogenesis and treatment options for oral cancer is warranted.

Partial epithelial to mesenchymal transition (EMT) is determined by cells exhibiting mesenchymal characteristics but without losing epithelial characteristics [6]. There is increasing evidence that this intermediate transition drives cancer cells invasion and metastasis, especially for HNSCC [7]. A study of single cell RNA sequencing pointed out partial EMT signature was associated with HNSCC invasion and node metastasis [8]. These clinical migration or invasion features, such as lymphovascular invasion (LVI), perineural invasion (PNI), or extracapsular extension (ECE), lead to disease progression in OSCC. Therefore, further investigation of different models of carcinogenesis and treatment options for oral cancer, especially focusing on partial EMT in OSCC, is warranted.

MicroRNAs (miRNAs) are noncoding RNA molecules that are 18–25 nucleotides long and can imperfectly bind to the 3′-UTR regions of target-gene mRNA and prevent its translation or promote its degradation, thereby inhibiting the cellular function of the target genes. The biological functions of miRNAs have been widely studied across several diseases, especially cancer. Some therapeutic strategies have accounted for the biological functions of miRNAs in cancer, such as by using miRNA inhibitors (antimiRs) to treat cancers with oncomir overexpression or by using miRNA mimics to treat cancers with low tumor-suppressive miRNA levels [9]. These technological developments piqued our interest in exploring new miRNAs in cancer and their potential applications in and implications for cancer therapy.

A previous study involved a global microarray analysis of miRNA expression in 40 oral squamous cell carcinoma (OSCC) specimens and their matched nontumorous epithelial tissues. MiR-455-5p was one of 84 miRNAs differentially expressed in this database [10]. Some studies have investigated the biological functions of miR-455-5p. In breast cancer, miR-455-5p can promote cancer cell invasion and migration by targeting its downstream gene *PDCD4* [11]. In lung cancer, miR-455-5p promotes cancer cell migration and invasion by inhibiting the tumor suppressor gene *SOCS3* [12]. In our previous study, we discovered that miR-455-5p could serve as an oncomir and promote OSCC cell proliferation [13]. When Smad3 binds to the promoter region of miR-455-5p, miR-455-5p expression can be upregulated by the transforming growth factor-β (TGF-β) pathway. Therefore, we hypothesized that miR-455-5p not only contributes to OSCC cell proliferation but also promotes OSCC cell migration and invasion. In the present study, we demonstrated that miR-455-5p could promote OSCC migration and invasion by regulating the EMT process. We also identified PDZK1-interacting protein 1 (*PDZK1IP1*) as a regulator of EMT in OSCC cells that could be suppressed by miR-455-5p. In addition, miR-455-5p and *PDZK1IP1* could participate in regulating partial EMT. Finally, we investigated whether *PDZK1IP1* could be used as a biomarker of OSCC in clinical practice.

## Methods

### Cell culture and 2.5D culture system

We cultivated SAS and HSC3 cells in minimum essential medium, OEC-M1 cells in RPM1-1640 medium (Gibco, Billings, MT, USA), and SCC4 and SCC9 cells in Dulbecco’s modified Eagle medium/nutrient mixture F-12 (Gibco, Billings, MT, USA). All the culture media were supplemented with 10% fetal bovine serum (Thermo Fisher Scientific) and 1 × antibiotic/antimycotic solution (Corning Cellgro, Manassas, VA). A 2.5D cell culture system was established in accordance with a protocol described in another study [14].

### Wound-healing and two-chamber transwell invasion assays

A wound-healing assay was performed to measure cell migration ability. Culture inserts (Ibidi, Martinscried, Germany) were used to construct two chambers. A total of 1 × 10^4^ OSCC cells were placed into one chamber and grew in a monolayer. Photos of the wound were taken immediately and 6 h after the inserts were removed. Cell migration was measured by using ImageJ software to calculate the wound closure area. Subsequently, a transwell invasion assay was conducted according to a previously described protocol [15].

### Cell mobility tracking

HSC-3 cells transfected with miR-455-5p-overexpressing plasmids, and control vectors were seeded in 6-well plates and tracked for 5 h. The position of each individual cell was captured through time-lapse microscopy with one shot taken every 2 min, and the migration patterns were analyzed using Nikon NIS-Elements AR software. A total of 28 cells in the control group and 39 cells in the miR-455-5p-overexpressing group were analyzed. The XY plots illustrate the positions of the cells during the tracking period.

### Western blotting

Western blotting was performed as previous described [15]. The primary anti-MAP17 antibodies (#ab156014, 1:1000) were purchased from Abcam (Cambridge, UK) and used for the Western blotting and immunohistochemical (IHC) analyses. The anti-E-cadherin (#610,182, 1:1000) and anti-N-cadherin (#610,920, 1:1000) antibodies were purchased from BD Transduction Laboratories (San Jose, CA, USA). The anti-glyceraldehyde 3-phosphate dehydrogenase (GAPDH; #2118, 1:10,000) antibodies were purchased from Cell Signaling Technology (Beverly, MA). The anti-vimentin antibodies (#sc-32322, 1:1000) were obtained from Santa Cruz Biotechnology (Santa Cruz, CA).

### Reverse transcription–quantitative polymerase chain reaction

The total RNA and complementary DNA were obtained as previous described [15]. GAPDH was used as the internal control to normalize gene expression. The following primer sequences were used in this study: 5′-CTGGTGGGAACAGATGGAA-G-3′ and 5′-TTGGAGCCACAGAGAAGGTT-3′ as the forward and reverse primers, respectively, for *PDZK1IP1* and 5′-TGAAGGTC-GGAGTCAACGGATT-3′ and 5′-CCTGGAAGATGG-TGATGGGATT-3’ as the forward and reverse primers, respectively, for GAPDH. The primer sequences for *PDPN*, *LAMC2*, and *LAMB3* were used as a previous study described [16]. The miR-455-5p quantitative PCR was conducted in accordance with a previously described protocol [13].

### Plasmid construction and lentivirus production

The pLe-miR-455-5p plasmids were donated by Dr. Shine-Gwo Shiah [13]. Short hairpin (sh) RNAs against *PDZK1IP1* were constructed. The target sequences of sh-*PDZK1IP1*-140 and sh-*PDZK1IP1*-185 were 5′-TTCCTGGTCCTCGTTGCAATC-3′ and 5′- TTGGAGCTC-AGACCGTGTCTA-3′, respectively. PLAS2w.Pneo vectors obtained from the National RNAi Core Facility (Academia Sinica, Taiwan) were used to induce MAP17 overexpression. Lentiviruses were manipulated in accordance with standard protocols (RNAi Core Laboratory of the Research Center of Clinical Medicine, National Cheng Kung University Hospital, Tainan, Taiwan).

### Luciferase reporter assay

The OSCC (OEC-M1) cells were seeded into 6-well plates. In the 3′-UTR luciferase reporter assays, the wild type and mutant 3′-UTR regions of the human *PDZK1IP1* gene were cloned into a pMIR-REPORT luciferase vector (Thermo Fisher Scientific). Smad-binding element-luciferase reporter (SBE4-Luc) plasmids were used for the Smad-binding activity assays. Cells cotransfected with a Renilla luciferase vector (pRL-SV40; Promega) at a DNA ratio of 100:1 were used as internal controls. The luciferase activity was measured using a dual-luciferase reporter assay system (Promega).

### Animal studies

For the xenograft metastatic assays, we injected 1 × 10^6^ stable luciferase-labeled oral cancer cells into the lateral tail vein of 6-week-old male NOD/SCID mice. At 7, 14, 21, 28, and 35 days after injection, the mice were examined using an IVIS imaging system (Caliper Life Science) to measure the luciferase signal intensity. After the experiment, the mice were euthanized, and their metastatic lung tumors were counted and photographed. The tissue samples were fixed in 10% formalin for hematoxylin and eosin staining. All the procedures were approved by the Institutional Animal Care and Use Committee of National Cheng Kung University (IACUC Approval No. 110062, Tainan, Taiwan).

### Nuclear and cytoplasmic fractionation

OEC-M1 cells were washed twice before cell scraping. Cytoplasmic lysis buffer (0.5 × Tris-buffered saline (TBS) with 0.1% NP-40 (Boston BioProducts) and 1 × protease inhibitors) was added in suspend cell pellet. Cells were lysed for 3 min on ice and then quick spun at 12,000 rpm for 10 s. Supernatant was collected as the cytosol fraction. Pellet was washed by 0.5 × TBS with 0.1% NP-40 twice and then lysed with nuclear lysis buffer (RIPA lysis buffer (Thermo Fisher Schientific) with 1 × protease inhibitor) for 10 min on ice. The lysates were spun for 10 min at 12,000 rpm and the supernatant was collected as the nuclear fraction. 1 × TBS with 0.1% SDS, 0.1% NP-40 and 1 × RIPA lysis buffer was used as lysis buffer for total cell lysate.

### Clinical samples and patient characteristics

All the formalin-fixed oral cancer tissues used this study were approved by the Institutional Review Board of National Cheng Kung University Hospital (A-ER-110–435). Available paraffin-embedded tissue blocks were obtained from patients with oral cancer diagnosed between January 2002 and December 2010 who had undergone curative surgery at National Cheng Kung University Hospital. No signs or events of distant metastases were identified at any of the initial diagnoses. The last follow-up date was in 2014.

### IHC analysis and quantification of IHC images

We obtained 3-μm-thick tissue sections from paraffin-embedded blocks. The IHC procedures were conducted using the BOND-MAX Autostainer (Leica Microsystems, United Kingdom) in accordance with the recommendations of the manufacturer. IHC staining was performed on consecutive sections from each archival tissue block after antigen retrieval in citrate buffer (pH 6.0) for 20 min. The sections were then incubated with primary antibodies against vimentin and MAP17 at room temperature for 30 min, with a BOND Polymer Refine Detection Kit (Leica Microsystems, United Kingdom) for 8 min, with 3,3-diaminobenzidine as the chromogen for 10 min, and with hematoxylin as the counterstain for 5 min. Two pathologists, who were blinded to the study, examined protein expression through a multiheaded microscope and evaluated the intensity and distribution to reach a consensus on the H-score, which was calculated as ΣPi (i + 1), where i represents the intensity of the stained tumor cells (0: negative, 1: weak positive, 2: moderate positive, and 3: strong positive) and Pi represents the percentage of stained tumor cells.

### Bioinformatic database analysis

To predict the *PDZK1IP1* 3′UTR binding sequence of miR-455-5p, we used TargetScan (http://www.targetscan.org/) and input the gene symbol (*PDZK1IP1*)*.* We located the predicted consequential pairing of *PDZK1IP1* 3′UTR and miR-455-5p on the list of poorly conserved sites for miRNA families conserved among vertebrates. We downloaded the Cancer Genome Atlas (TCGA) HNSCC data set and analyzed it using the UCSC Xena online exploration tool [17]. We then analyzed the relationship between clinical outcomes and *PDZK1IP1* expression by using the online KM Plotter tool (http://www.kmplot.com). The NKCU-OrCA-40TN (GSE37991) data set was used to analyze the correlation between *PDZK1IP1* expression and EMT-associated markers.

### Calculation of EMT and partial EMT scores

The EMT score was the sum of expression levels of several mesenchymal genes (*FN1*, *VIM*, *ZEB1*, *ZEB2*, *TWIST1*, *TWIST2*, *SNAI1*, *SNAI2*, and *CDH2*) minus the sum of expression levels of epithelial genes (*CLDN4*, *CLDN7*, *TJP3*, *MUC1*, and *CDH1*) [18]. The partial EMT score was the sum of expression levels of representative partial EMT-associated genes (*PDPN*, *LAMB3*, and *LAMC2*) [19].

### Statistical analysis

All the statistical data were analyzed using Student’s t test, a Mann–Whitney–Wilcoxon test, or a paired Wilcoxon signed-rank test. Correlations in gene expression were measured using the Pearson correlation coefficient. Kaplan–Meier estimation and the two-sided log-rank test were used to compare the survival rates of the patient groups. Cox proportional hazards model was used for multivariate analysis. Statistical analyses were conducted using MedCalc software (version 19.6), and the graphs were constructed using GraphPad Prism 9.0. A difference was considered statistically significant if the *p* value was < 0.05.

### Data availability

The datasets analyzed during the current study are available through Genomic Data Commons portal. The datasets of clinical patient samples are available from the corresponding author on reasonable request.

## Results

### MiR-455-5p promotes OSCC cell migration and invasion in vitro

**Fig. 1:**
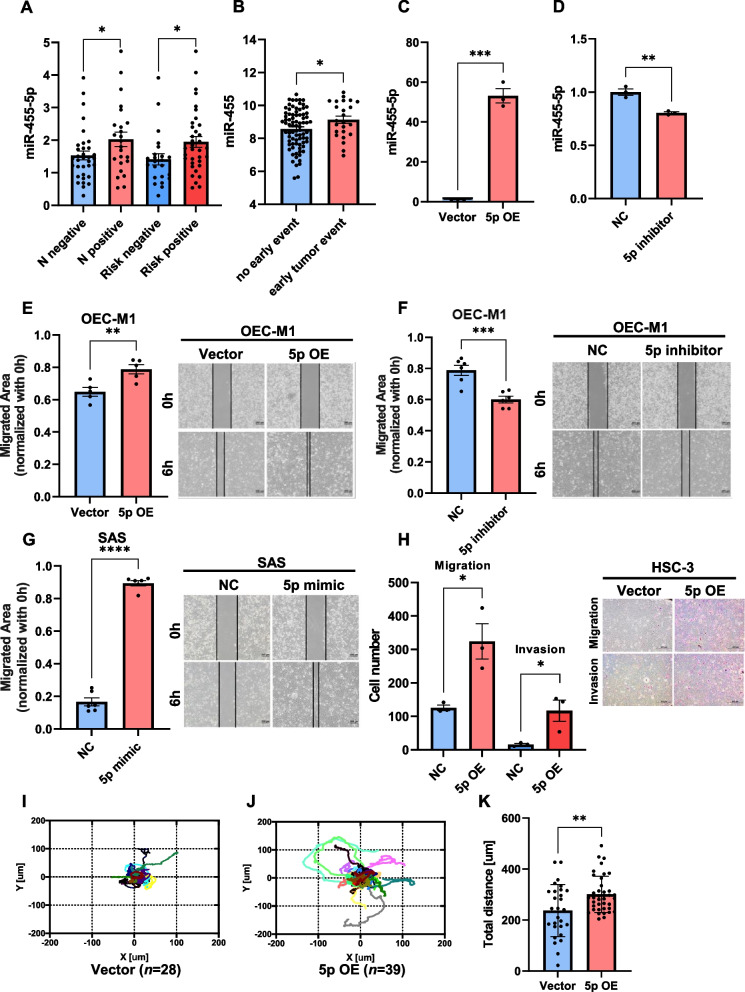
MiR-455-5p promotes OSCC cell migration and invasion. **A** MiR-455-5p expression in patients with and without node metastasis, or with and without clinical risk factors (perineural invasion, lymphovascular invasion, or extracapsular extension) in database of NKCU-OrCA-40TN. **B** MiR-455 expression in patients with and without early tumor event (new tumor event within one year) after initial treatment recorded in the TCGA head and neck carcinoma data set. **C** MiR-455-5p expression in OEC-M1 cells transfected with pLe-miR-455-5p plasmids and with vector control. **D** MiR-455-5p expression in OEC-M1 cells transfected with miR-455-5p inhibitor and controls. **E** Migration ability (determined by wound-healing assay) of OEC-M1 cells transfected with pLe-miR-455-5p plasmids and controls. **F** Migration ability (determined by wound-healing assay) of OEC-M1 cells transfected with miR-455-5p inhibitor and controls. **G** Migration ability of SAS cells transfected with miR-455-5p mimics (and controls) **H** Migration and invasion ability (determined by two-chamber transwell assay) of HSC-3 cells transfected with pLe-miR-455-5p plasmids and controls. I, J XY plots indicating the position of HSC-3 cells transfected with control vectors (**I**) or pLe-miR-455-5p (**J**) tracked for 5 h. **K** Total distance in XY plot (I, J). *p < 0.05, **p < 0.01, ***p < 0.001, ****p < 0.0001 by two-tailed Student’s *t* test

In our clinical cohort, the patients with OSCC and lymph node metastasis or clinical risk factors (namely LVI, PNI, or ECE) had relatively higher miR-455-5p expression levels (Fig. 1A). Data from the TCGA HNSCC data set (accessed through the Genomic Data Commons portal) also revealed that patients with early tumor event (new tumor event within one year) after initial treatment had higher miR-455-5p expression levels (Fig. 1B). MiR-455-5p expression not only affected OSCC patients’ overall survival in our clinical cohort (Fig. S1A) but also in TCGA HNSCC data set (Fig. S1B, accessed through online Kaplan–Meier plotter, kmplot.com/analysis/). We introduced lentivirus-mediated overexpression of miR-455-5p (pLe-miR-455-5p) in OEC-M1 cells (Fig. 1C). The migration ability of OEC-M1 cells with miR-455-5p overexpression was significantly higher than that of OEC-M1 cells with vector control in a wound-healing assay (Fig. 1E). Using antisense oligonucleotides to inhibit miR-455-5p expression in the OEC-M1 cells (Fig. 1D) weakened the cell’s ability to migrate (Fig. 1F). We induced the ectopic expression of miR-455-5p in SAS cells by using mimic oligonucleotides. The SAS cells with miR-455-5p overexpression exhibited higher migration ability in a wound-healing assay (Fig. 1G), and HSC-3 cells with miR-455-5p overexpression induced by the pLe-miR-455-5p plasmids exhibited not only a stronger migration ability in a wound-healing assay (Fig. S1C) but also a stronger migration and invasion ability in a two-chamber transwell invasion assay (Fig. 1H). In addition, HSC-3 cells with miR-455-5p overexpression exhibited higher motility and total migrated distance under time-lapse microscopy (Fig. 1I–K).

### *PDZK1IP1* is a potential target gene of miR-455-5p that regulates EMT in OSCC cells

**Fig. 2:**
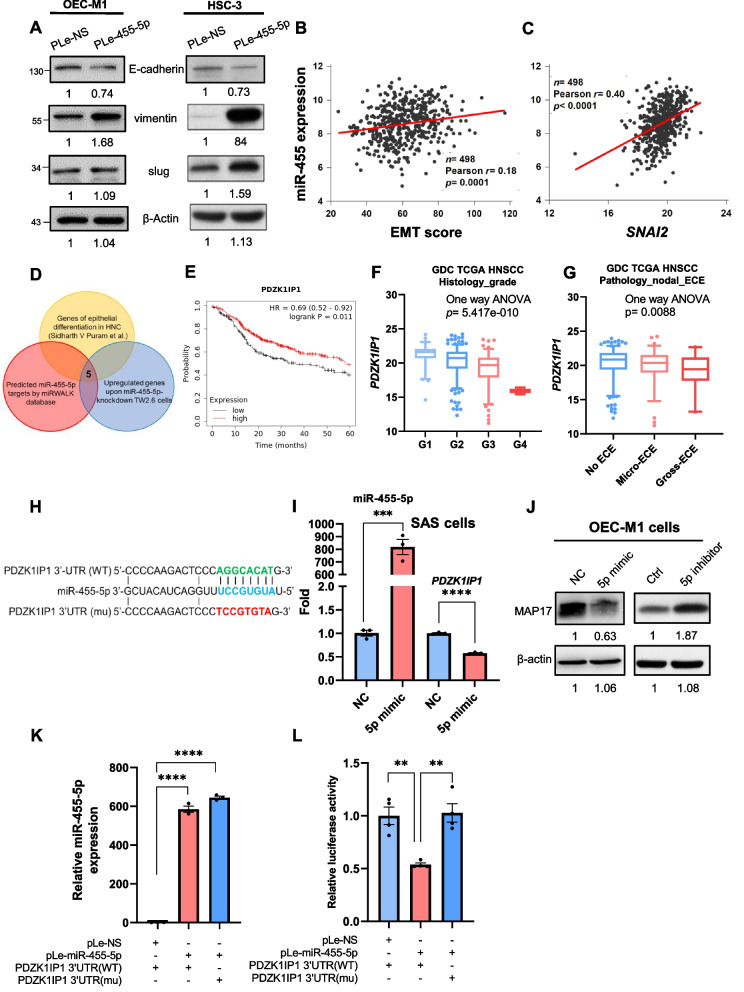
*PDZK1IP1* is a potential target gene of miR-455-5p. **A** Western blotting of OEC-M1 and HSC-3 cells transfected with pLe-miR-455-5p (and controls). β-actin was used as a loading control. **B**, **C** Association between the expression of miR-455 and EMT (**B**) score or *SNAI2* (**C**) in the TCGA head and neck squamous cell carcinoma database. **D** Venn diagram of predicted target genes of miR-455-5p. **E** Kaplan–Meier analysis revealed that high expression of *PDZK1IP1* is associated with higher rates of overall survival in patients with HNSCC. **F** *PDZK1IP1* expression of tumor samples with different histological differentiation. **G** *PDZK1IP1* expression of tumor samples with different patterns of pathologic nodal extracapsular spread, according to the TCGA database. ECE: extracapsular extension. **H** Schematic of *PDZK1IP1* 3′-UTR sequence containing miR-455-5p binding site and the mutant type of *PDZK1IP1* 3′-UTR sequence. **I** Relative miR-455-5p and *PDZK1IP1* mRNA expression in SAS cells transfected with miR-455-5p mimics and controls. **J** Western blotting revealed MAP17 protein expression in OEC-M1 cells transfected with miR-455-5p mimics and inhibitors (and controls). **K** Relative miR-455-5p expression in luciferase activity assay. **L** Wild type or mutant 3′-UTR of *PDZK1IP1* in luciferase activity assay. **p < 0.01, ***p < 0.001, ****p < 0.0001 by Student’s *t* test

TGF-β, a strong mediator of EMT, upregulates miR-455-5p through the targets against decapentaplegic (Smad) pathway [13]. In this study, OEC-M1 cells with miR-455-p overexpression exhibited more spindle cell morphology than did those without miR-455-p overexpression in the 2D cell culture (Fig. S2). OEC-M1 and HSC-3 cells with miR-455-5p overexpression by pLe-miR-455-5p exhibited higher expression of mesenchymal markers compared to it control cells, including vimentin and SLUG, and lower expression of the epithelial marker E-cadherin (Fig. 2A). Drawing on the data sets of TCGA HNSCC and NCKU-OrCA-40TN, we compared miR-455-5p expression by using EMT scores (calculated according to the study by Salt et al. [18]). EMT score was positively correlated with miR-455 expression (Fig. 2B and Fig. S3A). We also determined that the expression of miR-455 is positively correlated with *SNAI2*, which encodes an EMT transcription factor that plays a key role in HNSCC (Fig. 2C). In addition, using the ovarian cancer subtype data set [20], we determined that mesenchymal tumors exhibited elevated miR-455-5p expression (Fig. S3B). Therefore, we hypothesized that miR-455-5p regulates EMT in HNSCC. In the next step, we used the data sets for miRWALK (http://mirwalk.umm.uni-heidelberg.de), cDNA microarray of miR-455-5p-knockdown TW2.6 cells [13], and epithelial differentiation genes in the study by Puram et al. [8] to predict the target genes of miR-455-5p. In miRWALK prediction, we restricted the predicted genes included in more than 5 databases as the cutoff point. After combining these three data sets (Fig. 2D), we identified *PDZK1IP1* (encoded protein: MAP17) as a potential target gene (Fig. S3C). We also identified correlations among downregulated genes in the gene set of SARRIO_EPITHELIAL_MESENCHYMAL_TRANSITION_DN [21] and *PDZK1IP1*. The CSIOVDB is a database that can be used to determine the EMT scores of target genes [22]. We validated our results by using the CSIOVDB and discovered that *PDZK1IP1* expression was strongly negatively correlated with EMT scores (Fig. S3D). We used an online Kaplan–Meier plotter (kmplot.com/analysis/) to evaluate the effects of *PDZK1IP1* in HNSCC. The patients with high *PDZK1IP1* expression had longer overall survival times than did those with low *PDZK1IP1* expression (Fig. 2E). We evaluated *PDZK1IP1* expression by compiling data from the TCGA HNSCC data set by using UCSC Xena [17]. Tumors with histological poor differentiation exhibited lower *PDZK1IP1* expression (Fig. 2F). In addition, tumors with lymph node metastasis and gross ECE exhibited lower *PDZK1IP1* expression than those with micro-ECE and no ECE (Fig. 2G). The patients with pathologic N3 disease exhibited the lowest *PDZK1IP1* expression (Fig. S3E). Therefore, *PDZK1IP1* is a potential target gene of miR-455-5p and affects oral cancer outcomes by regulating EMT.

We used TargetScan (http://www.targetscan.org/) to determine whether the 3′-UTR region of *PDZK1IP1* contains a binding site for miR-455-5p. Figure 2H illustrates the sequence of predicted miRNA binding sites in the 3′-UTR region of *PDZK1IP1*. Next, we measured the miR-455-5p expression, *PDZK1IP1* mRNA expression, and MAP17 protein expression in various oral cancer cell lines. The SCC-15 and OC2 cell lines exhibited higher miR-455-5p expression than other cell lines, such as HSC-3, SAS, SCC-4, SCC-9, and OEC-M1 (Fig. S4A). As expected, the SCC-15 and OC2 cell lines had lower levels of *PDZK1IP1* mRNA expression than did the SAS, SCC-4, SCC-9, and OEC-M1 cell lines (Fig. S4B). *PDZK1IP1* mRNA (Fig. 2I, S4D) and MAP17 protein expression (Fig. 2J and Fig. S4C) in the OEC-M1 and SAS cell lines with miR-455-5p overexpression were significantly lower than those in their corresponding controls. Furthermore, OEC-M1 cells treated with miR-455-5p antisense oligonucleotides exhibited significantly higher MAP17 expression than did those left untreated (Fig. 2J and Fig. S4E). To verify the binding of miR-455-5p to the 3′-UTR region of *PDZK1IP1*, we cloned the wild type or mutant 3′-UTR region of *PDZK1IP1* into luciferase reporter plasmids (Fig. 2H), which were cotransfected with miR-455-5p-overexpression plasmids into OEC-M1 cells (Fig. 2K). The luciferase activity in the miR-455-5p-overexpression group was significantly lower than that in scrambled control (pLe-NS) group. However, the luciferase activity in the group with mutant 3′-UTR region of *PDZK1IP1* exhibited the opposite pattern (Fig. 2L). These results indicate that miR-455-5p suppresses *PDZK1IP1* by binding to the 3′-UTR region of *PDZK1IP1*.

### *PDZK1IP1* suppression promotes OSCC cell migration and metastasis by regulating the TGF-β signaling pathway

**Fig. 3:**
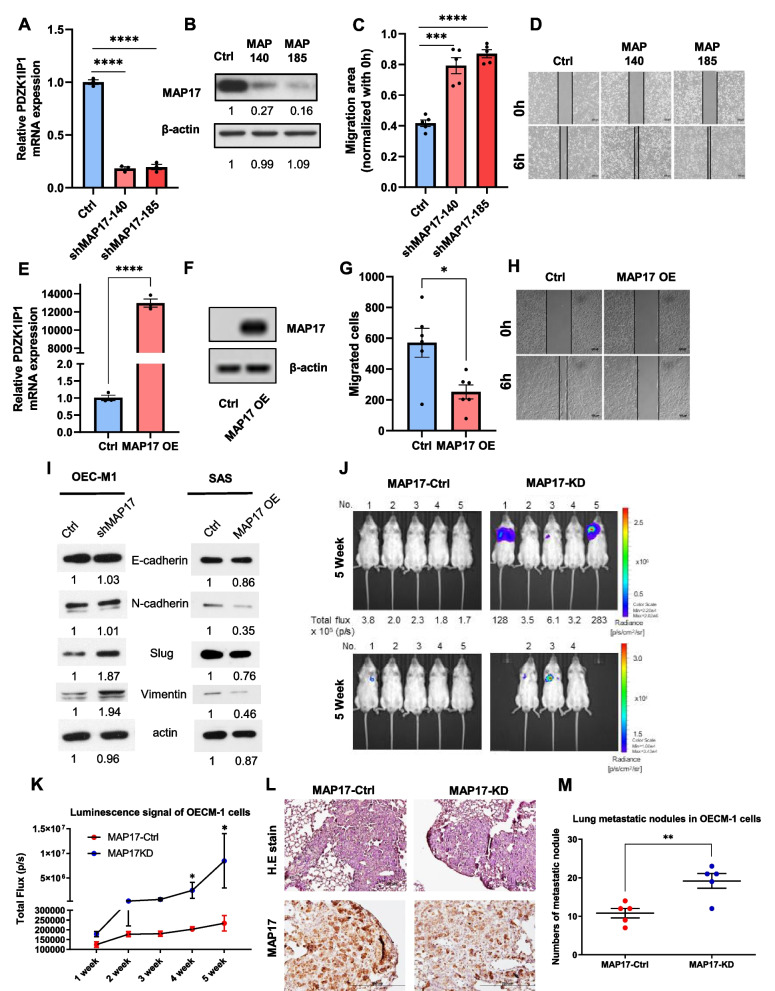
*PDZK1IP1* suppression promotes OSCC cell migration and metastasis in vitro and in vivo. **A-D** OEC-M1 cells transfected with shMAP17-140 and shMAP17-185 for silencing endogenous *PDZK1IP1* expression (and controls) (**A, B**) and their measured migration ability (**C, D**). **E–H** mRNA (**E**) and protein (**F**) expression of SAS cells transfected with *PDZK1IP1* overexpression plasmids and their migration ability, as determined through a wound-healing assay (**G, H**). **I** Western blotting of OEC-M1 cells transfected with shMAP17 and SAS cells transfected with *PDZK1IP1* overexpression plasmids. **J** In the animal model experiment, IVIS imaging was used to detect luciferase signals in the group with *PDZK1IP1*-knockdown OEC-M1 cells and the control group. Lower panel, the radiance range was reduced (from 2.2e4-2.8e6 to 1e4-3.4e4) to see low-intensity images of some mice. **K** Luciferase signals detected using an IVIS imaging system from week 1 to week 5. *p < 0.05 by Mann–Whitney U Test. **L** Metastatic lung nodules were retrieved after mice were euthanized. Hematoxylin and eosin staining was used to detect tumor parts and IHC staining was used to detect MAP17. **M** Calculated metastatic lung nodules after mice were killed. *p < 0.05, **p < 0.01, ***p < 0.001, ****p < 0.0001 by Student’s *t* test

We cultivated *PDZK1IP1*-knockdown OEC-M1 cells, namely shMAP17-140 and shMAP17-185 (Fig. 3A, B), which exhibited higher migration ability than that of the original OEC-M1 cell line with vector control in a wound-healing assay (Fig. 3C, D). *PDZK1IP1*-knockdown SCC-9 cells also exhibited higher migration ability (Fig. S4F, S4G). In addition, we cultivated SAS cells with *PDZK1IP1* overexpression by lentiviral transfection (Fig. 3E, F). The SAS cells with *PDZK1IP1* overexpression exhibited lower migration ability than did those with vector control (Fig. 3G, H). We further tested EMT markers in the *PDZK1IP1*-knockdown OECM-1 cells and *PDZK1IP1*-overexpressed SAS cells. The *PDZK1IP1*-knockdown OEC-M1 cells exhibited higher vimentin and SLUG expression than did the OEC-M1 cells with vector control, and the levels of N-cadherin, SLUG, and vimentin in the SAS cells with *PDZK1IP1* overexpression were lower than those in the SAS cells with vector control (Fig. 3I). To verify the effect of *PDZK1IP1* on OSCC migration in vivo, we cultivated *PDZK1IP1*-knockdown OEC-M1 cells with luciferase-labeling genes and performed intravenous tumor cell injection to observe potential lung metastasis. When the cells were examined using an in vivo imaging system (IVIS), the *PDZK1IP1* knockdown group exhibited higher lung metastatic ability with higher luminescence signals than did the control group (Fig. 3J, K). The metastatic lung nodules of the *PDZK1IP1* knockdown group exhibited less MAP17 protein expression (Fig. 3L). The *PDZK1IP1* knockdown group also had more metastatic lung nodules than did the control group (Fig. 3M).

**Fig. 4:**
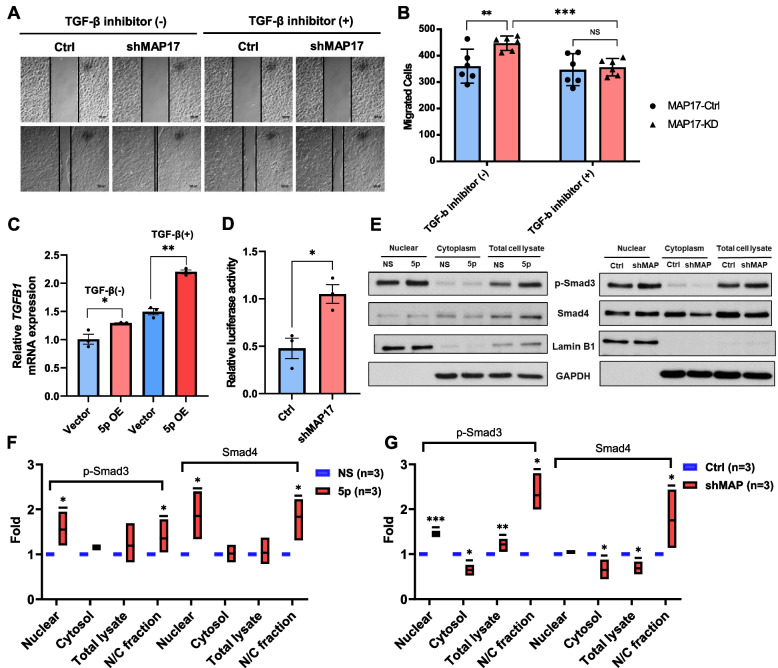
*PDZK1IP1* suppression promotes OSCC migration by regulating TGF-β signaling. **A, B** Wound-healing assays of OEC-M1 cells transfected with shMAP17 and controls, treated with TGF-β inhibitor or not. **C** *TGFβ1* mRNA expression of OEC-M1 cells transfected with pLe-miR-455-5p (or controls). The difference was increase after cells treated with TGF-β1 (10 ng/ml, #100–21, PeproTech). **D** Smad-binding element luciferase reporter assay of OEC-M1 cells transfected with shMAP17. **E** Western Blot analysis exhibited nuclear and cytoplasmic fractionation of phosphor-Smad3 (p-Smad3) or Smad4. **F-G** Box plots indicated fold change of p-Smad3 or Smad4 in nuclear and cytoplasmic fractionation of OEC-M1 cells transfected with pLe-miR-455-5p (or controls) (**F**) or transfected with shMAP17 (or controls) (**G**)

In our study, *PDZK1IP1* knockdown promoted OEC-M1 cell migration. However, the promoting effect of *PDZK1IP1* knockdown on OEC-M1 cell migration was attenuated after the cells were treated with TGF-β inhibitors (Fig. 4A, B). A previous study demonstrated that *PDZK1IP1* traps Smad4, thereby suppressing R-Smad phosphorylation, p-Smad3 and Smad 4 translocation to nucleus and TGF-β signaling [23]. We tested the TGF-β1 expression of OEC-M1 cells, and the result revealed that the OEC-M1 cells with miR-455-5p overexpression exhibited higher *TGFB1* mRNA expression than did the control cells (Fig. 4C). *PDZK1IP1* knockdown in OEC-M1 cells increased Smad-binding element–(CAGA)12-luciferase reporter activity (Fig. 4D). Our study also demonstrated that increase p-Smad3 and Smad4 translocation to nucleus as determined by nuclear/cytoplasmic fractionation in OEC-M1 cells with miR-455-5p overexpression (Fig. 4E, F). OEC-M1 cells with *PDZK1IP1* knockdown also exhibited more p-Smad3 and Smad4 nuclear translocation than did the control cells (Fig. 4E, G). These data indicate that miR-455-5p and *PDZK1IP1* regulate Smad binding activity and interfere with TGF-β signaling in OSCC cells, thereby regulating OSCC cell migration.

### MiR-455-5p expression and *PDZK1IP1* suppression induce collective migration by regulating partial EMT in OSCC cells

**Fig. 5:**
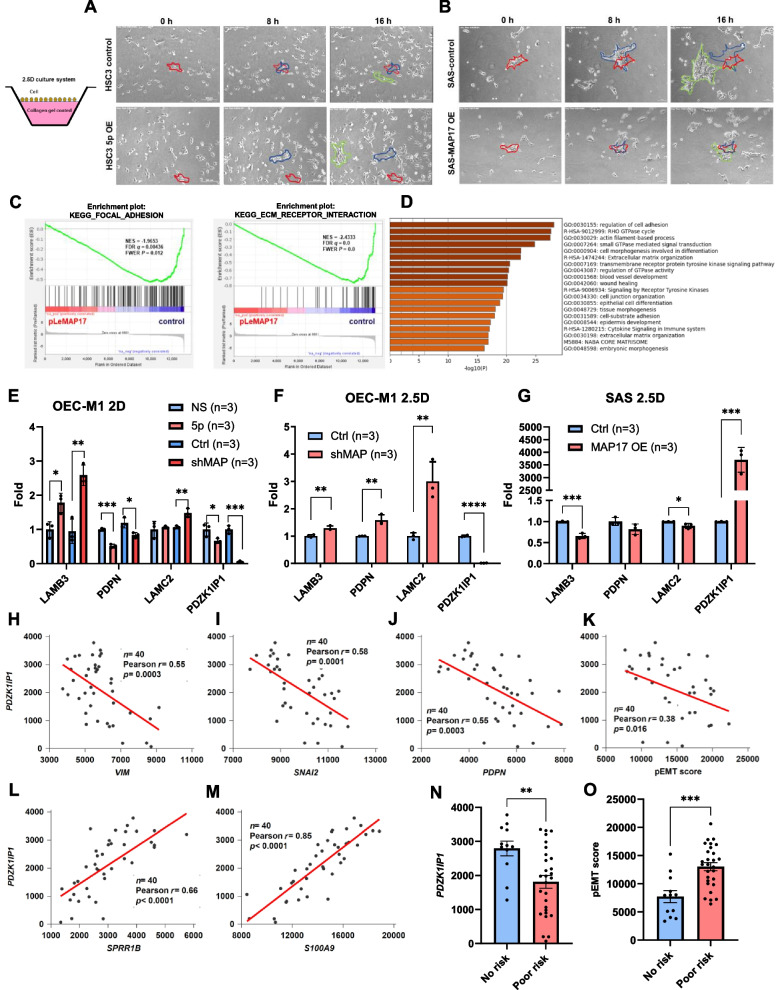
MiR-455-5p and *PDZK1IP1* regulate collective migration and partial EMT in OSCC cells. **A** Upper panel: HSC-3 control cells cultured in 2.5D culture medium. Lower panel: HSC-3 cells transfected with pLe-miR-455-5p in 2.5D culture medium. **B** Upper panel: SAS control cells exhibited higher migrative ability and collective cell movement in 2.5D cell culture. Lower panel: SAS cells transfected with *PDZK1IP1* overexpressed plasmid demonstrated low migrative ability and loss collective pattern of cell movement. (Red circle indicated tumor cluster at time point of 0-h, blue circle indicated tumor cluster at 8 h later and green circle indicated tumor cluster at 17 h later). **C** GSEA software analysis pointed out KEGG_FOCAL_ADHESIN and KEGG_ECM_RECEPTOR_INTERACTION gene sets. **D** RNA sequencing dataset from SAS cells with *PDZK1IP1* overexpression in 2.5D collagen-coated culture system was evaluated by online tool: metascape. Bar graph demonstrated GO enrichment analysis. **E** *LAMB3, PDPN, LAMC2 or PDZK1IP1* mRNA expression of OEC-M1 cells transfected with pLe-miR-455-5p (or controls) or shMAP17 (or controls) in 2D culture system. **F** *LAMB3*, *PDPN*, *LAMC2* or *PDZK1IP1* mRNA expression of OEC-M1 cells transfected with shMAP17 or control in 2.5D collagen-coated culture system. **G** *LAMB3, PDPN, LAMC2 or PDZK1IP1* mRNA expression of SAS cells transfected with MAP17 overexpression plasmids or control in 2.5D collagen-coated culture system. **H–M** Correlations between *PDZK1IP1* expression and *VIM* (**H**), *SNAI2* (**I**), *PDPN* (**J**)*,* partial EMT score (**K**)*, SPRR1B* (**L**) and *S100A9* (**M**) expression in the NKCU-OrCA-40TN data set. **N**. *PDZK1IP1* expression in patients with and without risk factors for recurrence (perineural invasion, lymphovascular invasion, and extracapsular extension) in the NKCU-OrCA-40TN data set. O Partial EMT scores of patients with and without risk factors for recurrence in the NKCU-OrCA-40TN data set. *p < 0.05, **p < 0.01, ***p < 0.001 by Student’s *t* test. Correlation *p* value was determined through Pearson correlation analysis

According to previous experimental results, miR-455-5p overexpression and *PDZK1IP1* suppression induce EMT in OSCC cells. We used a 2.5D cell culture platform and observed cell movement through time-lapse photography. In the 2.5D collagen-coated culture system, the movement of the HSC3 cells with vector control exhibited dispersed, single-cell migration (Fig. 5A, and Video S1A). Notably, the HSC3 cells with miR-455-5p overexpression tended to interact with nearby cells and exhibit collective migration patterns (Fig. 5A, and Video S1B). We also cultured SAS cells in the 2.5D collagen-coated culture system. In a previous study by Li et al. [24], SAS cells exhibited collective migration patterns in a 2.5D culture system (Fig. 5B and Video S2A). However, collective migration patterns (Fig. 5B and Video S2B) and predicted collective-migration gene profiles (Fig. 5C) were less common among the SAS cells with *PDZK1IP1* overexpression than among the control cells. Gene ontology analysis predicted that *PDZK1IP1* regulates collective migration (Fig. 5D).

A study involving single cell RNA sequencing revealed that partial EMT plays a key role in head and neck cancer [8]. Collective cell migration is induced by partial EMT signatures. In a subsequent study, researchers established the partial EMT score, which is the sum of the expression of representative markers of partial EMT-associated genes, such as *PDPN*, *LAMC2*, and *LAMB3* [19]. The study also revealed that partial EMT scores are associated with clinical outcomes in patients with oral cancer. In addition, the researchers identified some epithelial differentiation markers, such as *SPRR1B* and *S100A9*, that were negatively correlated with partial EMT scores [8]. Therefore, we used the expression levels of three partial EMT–associated genes (*PDPN*, *LAMB3*, and *LAMC2*) to calculate partial EMT scores. In the 2D culture system, OEC-M1 cells with miR-455-5p overexpression had higher *LAMB3* mRNA expression levels, and OEC-M1 cells with *PDZK1IP1* knockdown had higher *LAMB3* and *LAMBC2* mRNA expression levels (Fig. 5E). In addition, in the 2.5D collagen-coated culture system, the OEC-M1 cells with *PDZK1IP1* knockdown had higher *LAMB3*, *PDPN* and *LAMC2* mRNA expression levels (Fig. 5F), and the *LAMB3*, *LAMC2* mRNA expression levels of the SAS cells with *PDKZI1P1* overexpression were decreased (Fig. 5G). To consolidate these findings, we assessed the associations of *PDZK1IP1* expression with various EMT-associated genes by using a microarray database of NKCU-OrCA-40TN (GSE37991). We observed a significant positive correlation between the expression of *PDZK1IP1* and that of *CDH1* (Fig. S5A), as well as negative correlations between the expression of *PDZK1IP1* and that of genes encoding mesenchymal markers such as vimentin and SLUG (*VIM* and *SNAI2; *Fig. 5H and 5I)*.* Using this microarray data set, we calculated EMT scores according to the expression of various EMT-associated genes [18]. The results revealed a negative correlation between *PDZK1IP1* and EMT score (Fig. S5B), indicating that *PDZK1IP1* is a key regulator of the EMT process in OSCC cells. We also explored the expression of partial EMT-associated genes in HNSCC cells by the NKCU-OrCA-40TN (GSE37991) and TCGA database. We investigated the effects of partial EMT scores by using the NKCU-OrCA-40TN data set. The expression of *PDZK1IP1* was negatively correlated with that of partial EMT-associated genes (Fig. 5J for *PDPN*; Fig. S5C for *LAMC2*) and positively correlated with that of epithelial genes (Fig. 5L for *SPRR1B*; Fig. 5M for *S100A9*). *PDZK1IP1* expression was also negatively correlated with partial EMT scores (Fig. 5K). Patients with clinical risk factors (namely LVI, PNI, or ECE) exhibit lower *PDZK1IP1* expression (Fig. 5N) and have higher partial EMT scores than do patients without such risk factors (Fig. 5O). Drawing on the TCGA database, we determined that miR-455 expression was positively correlated with partial EMT-associated gene expression and partial EMT score (Fig. S5E and S5F for *PDPN* and *LAMC2*, respectively; Fig. S5G for partial EMT score) and that the expression of *PDZK1IP1* was strongly positively correlated with that of key epithelial differentiation markers (Fig. S5H for *SPRR1B*; Fig. S5I for *S100A9*). In addition, we determined that patients with high partial EMT scores had lower rates of recurrence-free survival (according to the NKCU-OrCA-40TN database; Fig. S5D). The patients with HNSCC who had high partial EMT scores had lower overall survival rates than did those with low partial EMT scores (according to the TCGA database; Fig. S5J).

To consolidate the finding that miR-455-5p regulates EMT through *PDZK1IP1*, we introduced overexpression of miR-455-5p and *PDZK1IP1* by pLe-miR-455-5p plasmids and *PDZK1IP1* overexpression plasmids (or control). We established OEC-M1 cells with negative control, stable OEC-M1 pLe-455-5p cells, or OEC-M1 pLe-455-5p cells with *PDZK1IP1* overexpression (Fig. S6A and S6B). As our previous results, OEC-M1 cells with miR-455-5p overexpression exhibited higher migration and invasion ability than that of OEC-M1 cells with vector control. However, the ectopic expression of *PDZK1IP1* in OEC-M1 pLe-455-5p cells rescued the promoting effects of miR-455-5p and leaded to lower migration and invasion ability compared to OEC-M1 pLe-455-5p cells (Fig. S6C for wound-healing assay and Fig. S6D for two-chamber transwell invasion assay). The ectopic expression of *PDZK1IP1* in OEC-M1 pLe-455-5p cells also suppressed the induction of EMT and partial EMT in OEC-M1 pLe-455-5p cells, leaded to more *CDH1*, less *VIM*, *LAMB3*, and *LAMC2* mRNA expression compared to OEC-M1 pLe-455-5p cells (Fig. S6E). These results indicated that miR-455-5p promoted OSCC cells migration, invasion, inducing EMT and partial EMT through *PDZK1IP1*.

### *PDZK1IP1* expression is negatively correlated with miR-455-5p expression and associated with more favorable prognoses in patients with OSCC

**Fig. 6:**
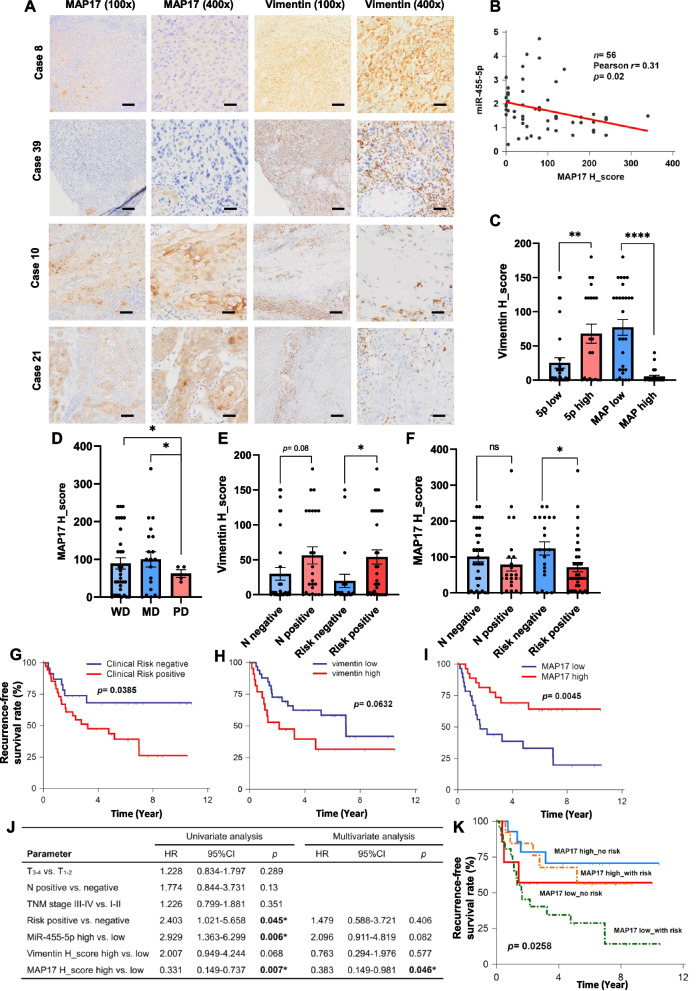
Clinical correlations among miR-455-5p, MAP17, vimentin, and survival outcomes. **A** IHC stain for vimentin and MAP17 in patients with high vimentin expression (case 8 and case 39) or with low vimentin expression (case 10 and case 21). Scale bar in 100x: 200 μm; in 400x: 50 μm. **B** Correlation between miR-455-5p expression and MAP17 H-score; *p* = 0.02 by Pearson correlation. **C** Vimentin H-scores of patients with high or low miR-455-5p expression, and with high or low MAP17 H-scores. **p < 0.01, ****p < 0.0001 by Student’s *t* test. The expression levels of miR-455-5p, MAP17, and vimentin were divided into high and low groups according to their respective mean values or mean H-scores in the cohort. **D** MAP17 H-scores of patients with different histological differentiation. (WD: well-differentiation; MD: moderate-differentiation; PD: poor-differentiation) **E** Vimentin H-scores of patients with node metastasis or no node metastasis, and with clinical risk factors or no clinical risk factors. **F** MAP17 H-scores of patients with node metastasis or no node metastasis, and with clinical risk factors or no clinical risk factors. **G-I** Kaplan–Meier survival curve. Recurrence-free survival of patients with clinical risk factors or no risk factors (**G**), high or low vimentin expression (**H**) and high or low MAP17 expression (**I**). The *p* value was determined using a Mantel–Cox log-rank test. **J** Multivariate Cox proportional hazards model for recurrence-free survival. **K** Kaplan–Meier survival curve of patients with MAP17 low (or high) and clinical risk factors (or no risk factors)

Our previous study investigated miR-455-5p expression in OSCC specimens and demonstrated that patients with higher miR-455-5p expression had lower disease-free survival rates [13]. The cohort enrolled patients with oral cancer who had undergone curative surgery at National Cheng Kung University. Some patients with clinical risk factors for recurrence received adjuvant radiotherapy or concurrent chemo-radiotherapy. We further tested the expression of vimentin and MAP17 proteins through IHC staining. In clinicopathological characteristics, we determined that the expression level of miR-455-5p was positively correlated with the N stage of OSCC patients, and patients with low MAP17 expression exhibited more lymph node metastasis, LVI, or clinical poor risk factors for recurrence (Supplementary Table 1). In most cases, tumor samples from patients with high vimentin expression exhibited low MAP17 expression (Fig. 6A, case 8 and case 39) and samples from patients with low vimentin expression exhibited high MAP17 expression (Fig. 6A, case 10 and case 21). MAP17 expression (degree indicated by H-score) and miR-455–5-p expression were negatively correlated (Fig. 6B). The patients with high miR-455-p expression exhibited higher vimentin expression, and the patients with high MAP17 expression had significantly lower vimentin expression (Fig. 6C). We further evaluated the effects of vimentin and MAP17 on clinical outcomes. Patients with poor differentiation OSCC exhibited low MAP17 expression (Fig. 6D). This result was consistent with TCGA data set (Fig. 2F). Patients with node metastasis or clinical risk factors (namely LVI, PNI, or ECE) exhibited more vimentin expression than node negative or without clinical risk factors (Fig. 6E). In addition, patients with clinical risk factors exhibited lower MAP17 expression than without clinical risk factors (Fig. 6F). The patients with clinical risk factors or higher vimentin expression in tumor tissues had lower rates of recurrence-free survival than did those without clinical risk factors or with low tumor vimentin expression (Fig. 6G, H). The patients with higher MAP17 expression had higher rates of recurrence-free survival (Fig. 6I). To address the impact of MAP17 in our cohort, we established a multivariate Cox proportional hazards model. This model indicated MAP17 was an independent prognostic factor for recurrence-free survival (Fig. 6J). Kaplan–Meier plotter analysis also demonstrated patients with clinical risk factors and low MAP17 expression had worse clinical outcomes than patients with clinical risk factors but with high MAP17 expression (Fig. 6K). Besides, patients with high miR-455-5p expression and low MAP17 expression had the worst clinical outcome in recurrence-free survival (Fig. S6F).

## Discussion

Studies of the effects of miRNAs on cancer-associated mechanisms have focused on cell proliferation, resistance to cell death, metastasis induced by the EMT process, and angiogenesis [25]. Evidence of the clinical applications of miRNAs in cancer treatment has been accumulating. Circulating miRNAs could serve as cancer biomarkers, and miRNA panels could be used for cancer risk prediction [26, 27]. Several early-phase clinical trials involving miRNA detection have been conducted [28]. In another study, researchers attempted to resolve human disease on the basis of the results of previous miRNA studies [9]. Therefore, exploring new miRNAs or alternative functions of miRNAs is key to advancing cancer therapy. Some studies have investigated the roles of miRNAs in the carcinogenesis of OSCC. MiR-326 and miR-410 could target Wnt7B and activate the Wnt signaling pathway [10]. MiR-320, which regulates tumor angiogenesis by targeting neuropilin 1, is decreased in vascular endothelial cells of OSCC tissues [29]. MiR-376c may target the RUNX2/Activin-A axis; therefore, downregulation of miR-376c may promote lymph node metastasis in OSCC [30]. MiR-450a may promote the invasion ability of OSCC cells by inhibiting TMEM182 expression [31]. In the present study, we demonstrated that miR-455-5p affects the clinical outcomes of patients with OSCC. Patients with OSCC and clinical risk factors (LVI, PNI, or ECE) exhibit high miR-455-5p expression, which affects their chances of disease-free survival. In addition to promoting cell proliferation, miR-455-5p promotes OSCC cell migration and invasion and induces EMT. Furthermore, we identified *PDZK1IP1* as a target gene of miR-455-5p that may inhibit migration and affect EMT in OSCC cells. MiR-455-5p overexpression suppressed *PDZK1IP1* expression in OSCC cells in vitro.

MAP17, which is encoded by *PDZK1IP1* and also called DD96 or SPAP, is a small (17-kDa) non-glycosylated transmembrane protein located in the plasma membrane and Golgi apparatus [32]. MAP17 expression is limited in some normal human tissues, such as the proximal tubular epithelial cells of the kidney. However, diffuse expression has been documented not only in renal cell carcinoma but also in some epithelial malignancies, namely colon, breast, and lung cancer [33, 34]. In normal human tissues, MAP17 acts as a cargo protein by interacting with NaPi-IIa transporter in renal cells or PDZK1 in hepatocytes [35, 36]. However, several studies have reported that MAP17 may play an important role in carcinogenesis. MAP17 expression in patients with advanced neoplasms, such as colon adenocarcinoma, is higher than that in patients with premalignant changes or normal colon epithelia [31]. Previous studies reported that MAP17 facilitates the production and enhances the tumorigenic properties of reactive oxygen species (ROS) [37]. By inducing ROS creation, MAP17 could serve as an intersection between cancer and inflammatory disease, further affecting the tumor microenvironment [38, 39]. In addition, by inhibiting p21/WAF1 induction, MAP17 can prevent tumor necrosis factor (TNF)-induced G1 arrest in cancer cells [40]. MAP17 sequestrates NUMB, a protein that traps NICD and prevents its translocation to nuclei, thereby activating the Notch pathway [41]. In breast cancer cells, MAP17 can be carried by exosomes and released as cargo to promote the horizontal propagation of EMT, cancer stemness, and metastasis [42].

Although some studies have reported that *PDZK1IP1* has tumorigenic functions, others have reported that it has tumor-suppressive abilities. In patients with laryngeal cancer, *PDZK1IP1* expression is correlated with higher rates of laryngoesophageal dysfunction–free and overall survival [43]. One study revealed that *PDZK1IP1* and *SGLT1* expression are key prognostic biomarkers for patients with cervical cancer receiving cisplatin plus radiotherapy. In that study, although *PDZK1IP1* was identified as a marker of carcinogenesis that increases ROS generation through SGLT1, the patients with high MAP17 had a more favorable responses to treatments that boosted oxidative stress [44]. In patients with lung adenocarcinoma, *PDZK1IP1* expression may be used to predict a patient’s response to cisplatin, carboplatin, EGFR inhibitors, or bortezomib, which is clinically beneficial for patients with lung cancer [45]. TGF-β signaling is negatively regulated by *PDZK1IP1*, which can trap Smad4 proteins, thereby affecting the formation of R-Smad/Smad4 complexes [23]. Our study also identified *PDZK1IP1* as a favorable prognostic factor in OSCC. *PDZK1IP1* knockdown promoted OSCC migration in vitro and metastasis in vivo. *PDZK1IP1* suppressed TGF-β signaling in a Smad-binding element reporter assay, which is consistent with the results of a study by Ikeno [23]. In patients with clinical OSCC, miR-455-5p expression was negatively corelated with MAP17 H-score when assessed using IHC staining. We also verified that patients with relatively high MAP17 expression in tumor tissues had relatively high rates of recurrence-free survival and that MAP17 affects cell migration and invasion, metastasis, and clinical outcomes in patients with OSCC. MAP17 was also serve as an independent factor that impacted outcomes of patients, especially for patients with clinical risk factors for recurrence.

Lymph node involvement and distant metastasis contribute to poor clinical outcomes in patients with HNSCC. LVI, PNI, and ECE are major risk factors for recurrence after surgery. Therefore, determining the mechanisms that underlie cell migration and invasion is crucial for oral cancer treatment. Studies of the biological processes associated with tumor migration and invasion have focused on cell adhesion, angiogenesis, lymphangiogenesis, and EMT [46, 47]. EMT, a cellular process through which cancer cells can acquire migration and invasion abilities, may be associated with cancer stemness and resistance to therapy [48, 49]. The gene signature of EMT is related to elevated levels of immune checkpoint molecules and associated with poor prognosis in patients with HNSCC [50]. Recent studies suggest that EMT is a progressive step in cellular change rather than a bipolar mode of cellular behavior. Therefore, EMT score, calculated according to the expression of different EMT-associated genes, may be utilized in clinical applications to predict patients’ responses to therapy [18]. The full EMT process is not easily observed during HNSCC progression. Several studies theorized that partial EMT, rather than full EMT, plays a key role in HNSCC progression [7, 51]. Partial EMT is spatially localized at the leading edges of HNSCC tumors, which it is crucial for HNSCC invasion and migration [8]. Partial EMT is related to collective migration because epithelial adhesion markers expressed by cells in partial EMT may promote cell–cell adhesion [6]. Collective migration has been determined to play a major role in cancer migration, invasion, and metastasis [52]. Cancer cells with collective movement patterns are more capable of invading surrounding tissues and metastasizing distantly. In addition, some studies have also reported that circulating tumor microemboli exhibit great resistance to cytotoxic agents [53]. In oral cancers, snail-induced claudin-11 promotes collective migration, thereby promoting cancer invasion and tumor cluster circulation [24]. Therefore, the biological functions of EMT, especially partial EMT, play pivotal roles in HNSCC. A study of single cell transcriptomic analysis pointed out gene sets of six meta-signatures, including cell cycle, partial EMT, epithelial differentiation, stress, and hypoxia [8]. *SNAI2*, *PDPN*, *LAMB3*, and *LAMC2* serves as important partial EMT-associated genes and could be applied to represent partial EMT transition [16, 54]. The sum of expression of *PDPN*, *LAMB3*, and *LAMC2* could be calculated as partial EMT score [19]. In addition, some important genes listed in the gene set of epithelial differentiation, such as *SPRR1B* and *S100A9*, could serve as markers of well-differentiated epithelial cells [8]. Studies also pointed out *SPRR1B* represented as a squamous differentiation marker and S100A9 is directly associated with cellular differentiation in HNSCC [55, 56]. The data of single cell transcriptomic analysis also demonstrated epithelial differentiation expression program is negative correlated with partial EMT program in HNSCC [8].

**Fig. 7:**
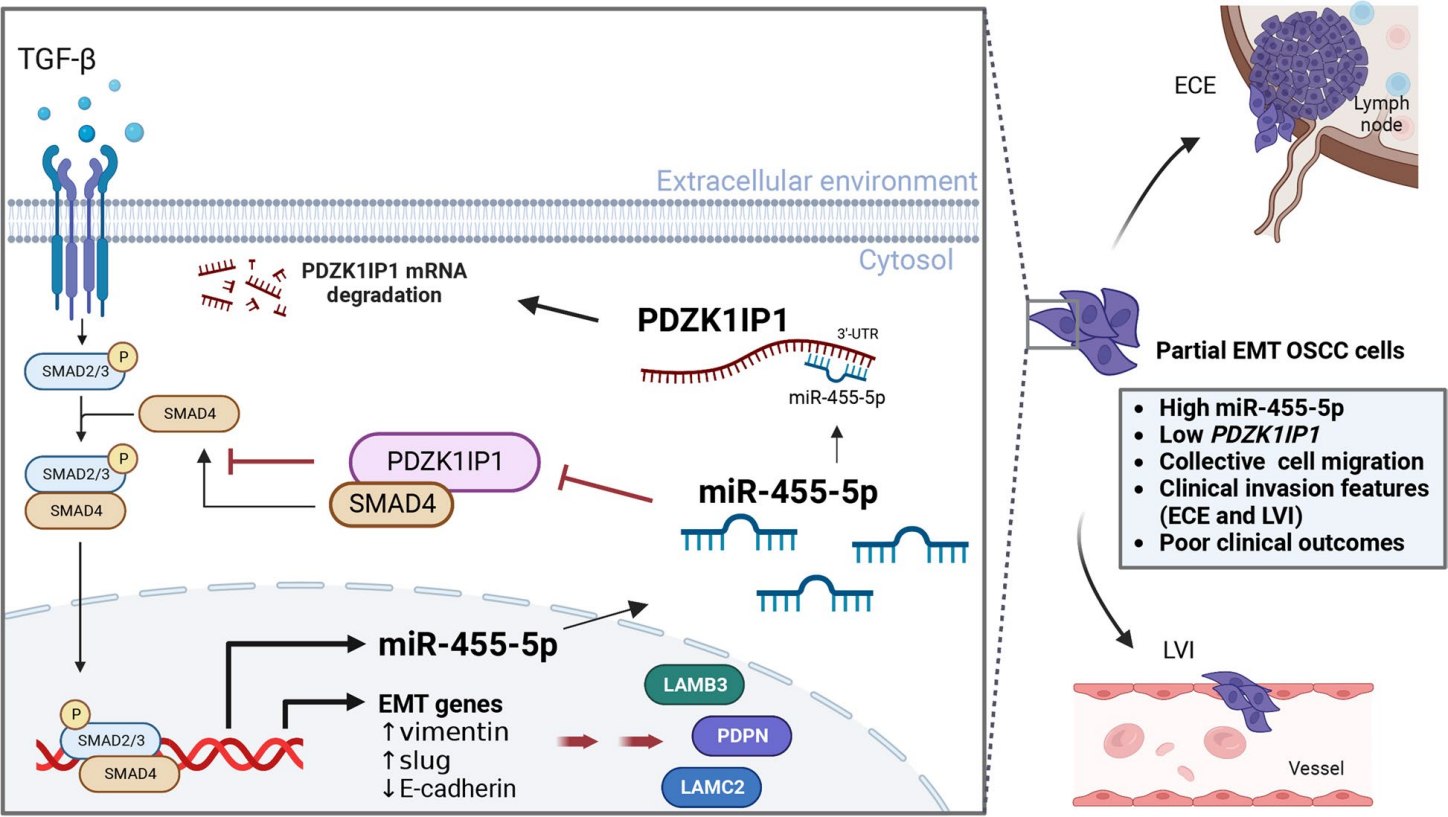
Schematic illustrating how OSCC migration is promoted by the miR-455-5p-*PDZK1IP1* axis. Positive feedback occurs when *PDZK1IP1* suppression facilitates TGF-β signaling and then induces miR-455-5p expression. MiR-455-5p upregulation and *PDZK1IP1* suppression induce EMT and partial EMT, which contribute to collective migration, invasion, and poor clinical outcomes in OSCC. (*ECE* Extracapsular extension, *LVI *Lymphovascular invasion)

Few studies have explored the effect of *PDZK1IP1* expression on EMT. One study by García-Heredia’s et al. revealed that breast cancer cell lines exhibiting MAP17 overexpression had higher *SNAI1* and *CDH2* expression levels and lower *CDH1* expression levels, indicating that EMT can be induced through activation the Notch pathway [42]. However, on the basis of our analysis of the SARRIO_EPITHELIAL_MESENCHYMAL_TRANSITION_DN data set, *PDZK1IP1* expression is downregulated in cells undergoing EMT [21]. Our analysis of data from the CSIOVDB also revealed that *PDZK1IP1* expression is negatively corelated with EMT scores [22]. In addition, TGF-β is a strong EMT regulator and can be suppressed by *PDZK1IP1* [23]. In our study, *PDZK1IP1* suppression was associated with higher levels of SLUG and vimentin, and *PDZK1IP1* overexpression was associated with lower levels of N-cadherin, SLUG, and vimentin in OSCC cell lines. These data indicate that *PDZK1IP1* suppresses EMT in OSCC. SLUG is involved in wound healing and in partial EMT of HNSCC [8, 57]. Based on the data set of single-cell transcriptomic analysis in HNSCC [8], we determined that *PDZK1IP1* is a crucial factor for epithelial differentiation and could serve as a regulator of partial EMT. Collective cell migration, a specific cell movement pattern related to partial EMT, could be induced by miR-455-5p and inhibited by *PDZK1IP1* in OSCC cells. MiR-455-5p overexpression or *PDZK1IP1* suppression could induce the expression of partial EMT-associated genes, such as *PDPN*, *LAMB3*, or *LAMC2*, in 2D or 2.5D cell culture. Analysis of NKCU-OrCA-40TN (GSE37991) and TCGA HNSCC data sets revealed that positive correlation between miR-455 and partial EMT-associated genes and partial EMT score. *PDZK1IP1* was also positively correlated with epithelial differentiation-associated genes and negatively correlated with partial EMT score. Patients with OSCC and risk factors for recurrence exhibit lower *PDZK1IP1* expression and have higher partial EMT scores than do those without such risk factors. Therefore, we determined that *PDZK1IP1* could suppress OSCC cell migration and metastasis by regulating partial EMT. We also demonstrated that *PDZK1IP1* expression is negatively correlated with vimentin expression in clinical OSCC tissue samples. Importantly, our study identified *PDZK1IP1* as a target gene of miR-455-5p (Fig. 7). *PDZK1IP1* could be an important independent predictive marker for clinical outcomes in OSCC. In the future, miR-455-5p inhibitors could be considered for use in the treatment of patients with OSCC at high risk for recurrence.

## Conclusions

In summary, our results indicate that miR-455-5p suppresses *PDZK1IP1* expression and mediates OSCC progression. MiR-455-5p and *PDZK1IP1* may therefore serve as key biomarkers and be involved in regulating partial EMT in OSCC cells. *PDZK1IP1* expression may also serve as an independent factor that impacts outcomes in patients with clinical risk factors for recurrence.

## Supplementary Information

**Additional file 1.**

**Additional file 2.**

**Additional file 3.**

**Additional file 4.**

**Additional file 5.**

## Data Availability

All data generated or analyzed during this study are included in this published article and its supplementary information files.

## Abbreviations

HNSCC: Head and neck squamous cell carcinoma
OSCC: Oral squamous cell carcinoma
EMT: Epithelial-to-mesenchymal transition
EGFR: Epidermal growth factor receptor
LVI: Lymphovascular invasion
PNI: Perineural invasion
ECE: Extracapsular extension
