# Supplementary material for: MiR-455-5p suppresses PDZK1IP1 to promote the motility of oral squamous cell carcinoma and accelerate clinical cancer invasion by regulating partial epithelial-to-mesenchymal transition
Source: J Exp Clin Cancer Res. 2023 Feb 3;42:40. doi: 10.1186/s13046-023-02597-1 (PMC9896797; doi:10.1186/s13046-023-02597-1)
Supplement: Supplementary file 1 — Additional file 1. [file 13046_2023_2597_MOESM1_ESM.pdf]

Supplementary information

MiR-455-5p suppresses *PDZK1IP1* to promote the motility of oral squamous cell carcinoma and accelerate clinical cancer invasion by regulating partial epithelial-to-mesenchymal transition

Sheng-Yen Hsiao, Shang-Mei Weng, Jenn-Ren Hsiao, Yi-Ying Wu, Jia-En Wu, Chia-Hao Tung, Wan-Lin Shen, Shu-Fang Sun, Wen-Tsung Huang, Cheng-Yao Lin, Shang-Hung Chen, Tse-Ming Hong, Yuh-Ling Chen, Jang-Yang Chang

Corresponding Authors: Tse-Ming Hong, tmhong@ncku.edu.tw, Yuh-Ling Chen, Email: yuhling@ncku.edu.tw, and Jang-Yang Chang, Email: jychang@nhri.edu.tw.

Supplementary information includes Supplementary Figure legends, 6 Supplementary Figures, 4 Supplementary Videos, and 1 Supplementary Table.

Supplementary Figure Legends

Figure S1. A Kaplan–Meier overall survival curve of clinical patients with miR-455-5p low (5p low) or high (5p high). **B** In TCGA data set, Kaplan–Meier analysis revealed that high expression of miR-455 is associated with lower rates of overall survival in patients with HNSCC. **C** Wound-healing assay of HSC-3 cells transfected with pLe-miR-455-5p plasmid (or controls). $**p < 0.01$ by two-tailed Student's t test.

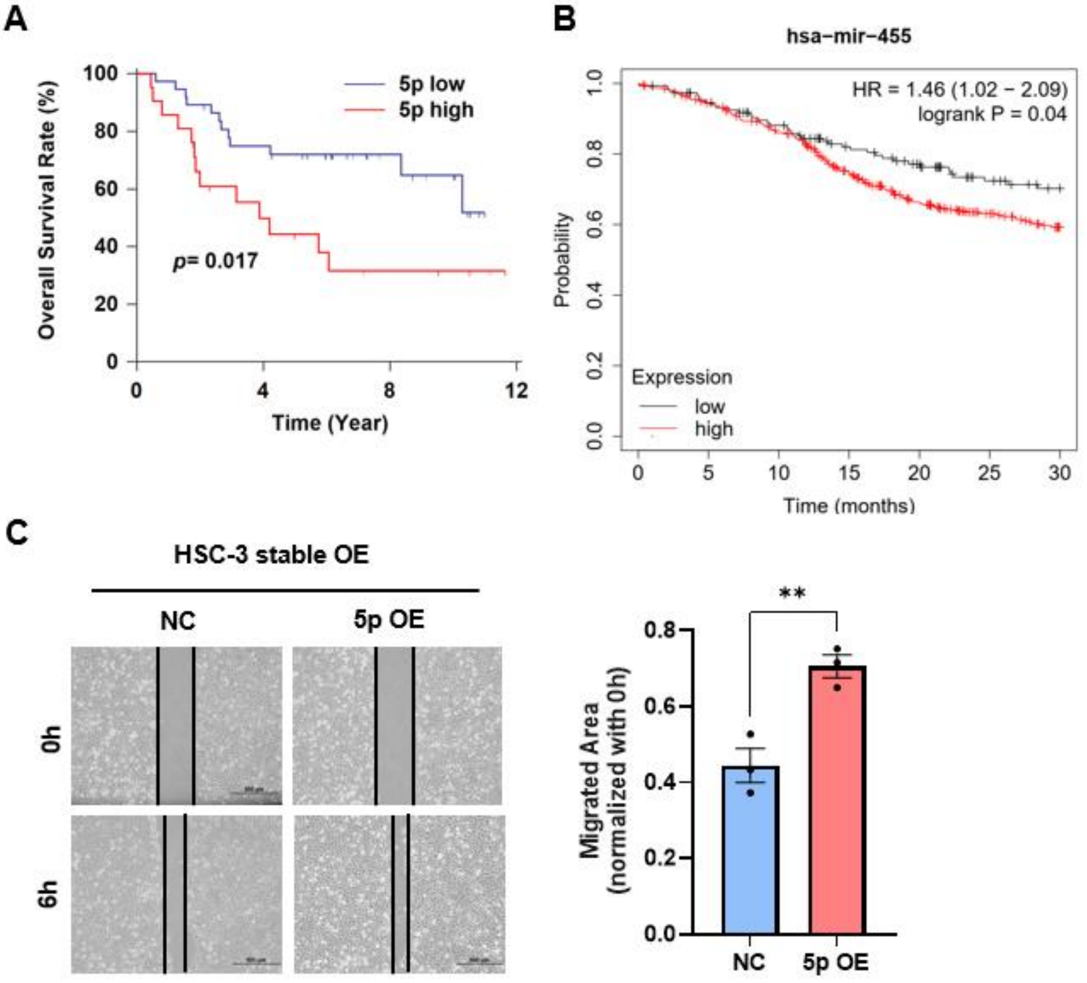

Figure S2. MiR-455-5p overexpression induced spindle cell morphology in OEC-M1. OEC-M1 cells transfected with pLe-miR-455-5p or miR-455-5p mimics demonstrated more cells with spindle cell morphology. Red quadrangles pointed out the area in upper panel. Bar plots demonstrated the difference in the counts of cells with spindle morphology per 100 cell counts between the two groups. *$p < 0.05$, ***$p < 0.001$ by two-tailed Student's t test.

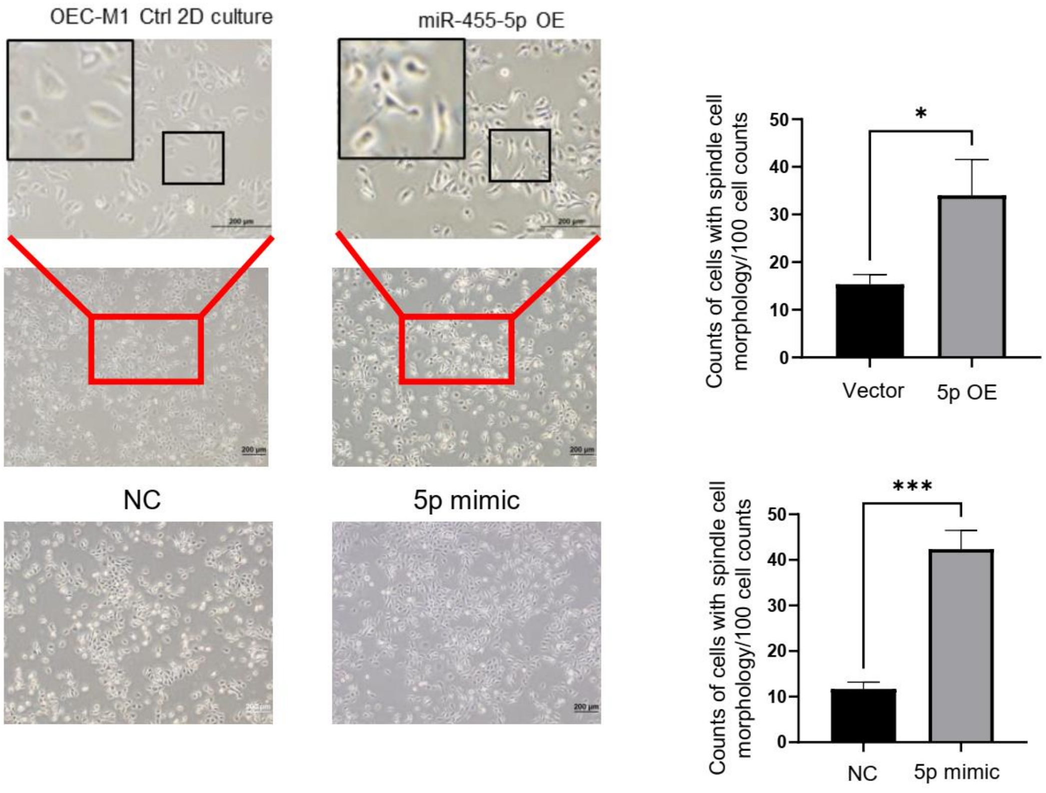

Figure S3. A The correlation between miR-455-5p and EMT score in NCKU-OrCA-40TN database. **B** Expression of miR-455-5p in non-mesenchymal and mesenchymal subtype from TCGA ovarian cancer database. **C** The list of predicted genes by Venn diagram in Fig. 2D. The table also demonstrated the survival outcome in KM plotter (kmplot.com/analysis/) and spearman correlation with EMT score in CSIOVDB database of individual genes. **D** The correlation between *PDZK1IP1* and EMT score in CSIOVDB database. **E** Expression of *PDZK1IP1* in different pathologic lymph node stages from TCGA head and neck cancer database.

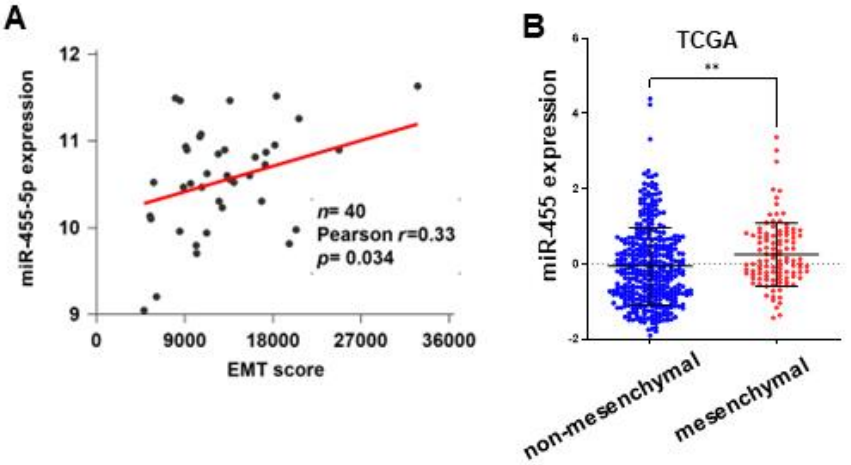

C

Gene	Survival in KM plotter	Spearman correlation with EMT score (Rho)
PDZK1IP1	good	-0.498330
OVOL1	good (n/s)	-0.240235
TMPRSS11E	good	-0.122355
LYPD3	good	-0.115427
CAST	good	0.005949

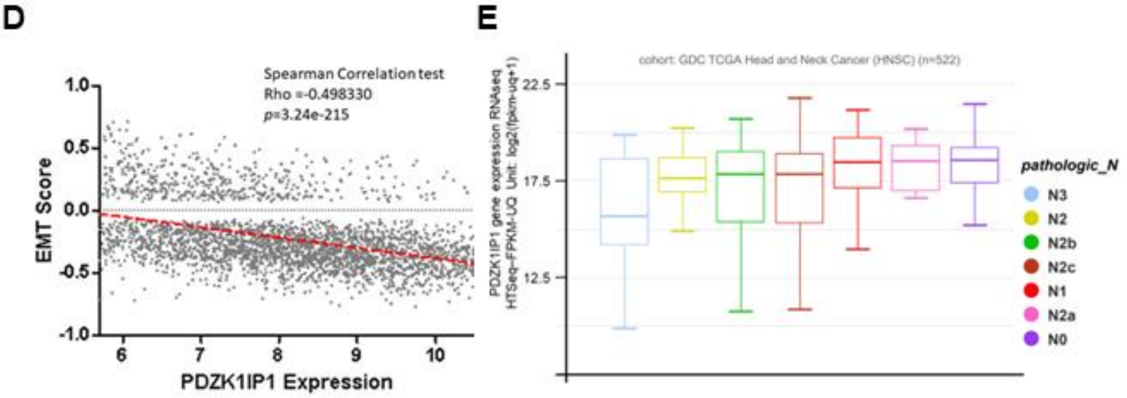

Figure S4. A MiR-455-5p expression in different OSCC cell lines. **B** *PDZK1IP1* mRNA expression in different OSCC cell lines. **C** Western blot analysis exhibited MAP17 expression of SAS cells transfected with miR-455-5p mimic or controls. **D, E** *PDZK1IP1* mRNA expression of OEC-M1 cells transfected with miR-455-5p mimic or inhibitor. **F, G** SCC-9 cells transfected with shMAP17 to silencing endogenous *PDZK1IP1* (**G**), and migration ability was measured by wound-healing assay (**F**). ***p*< 0,01, ****p*< 0.001, *****p*< 0.0001 by two-tailed Student's *t* test.

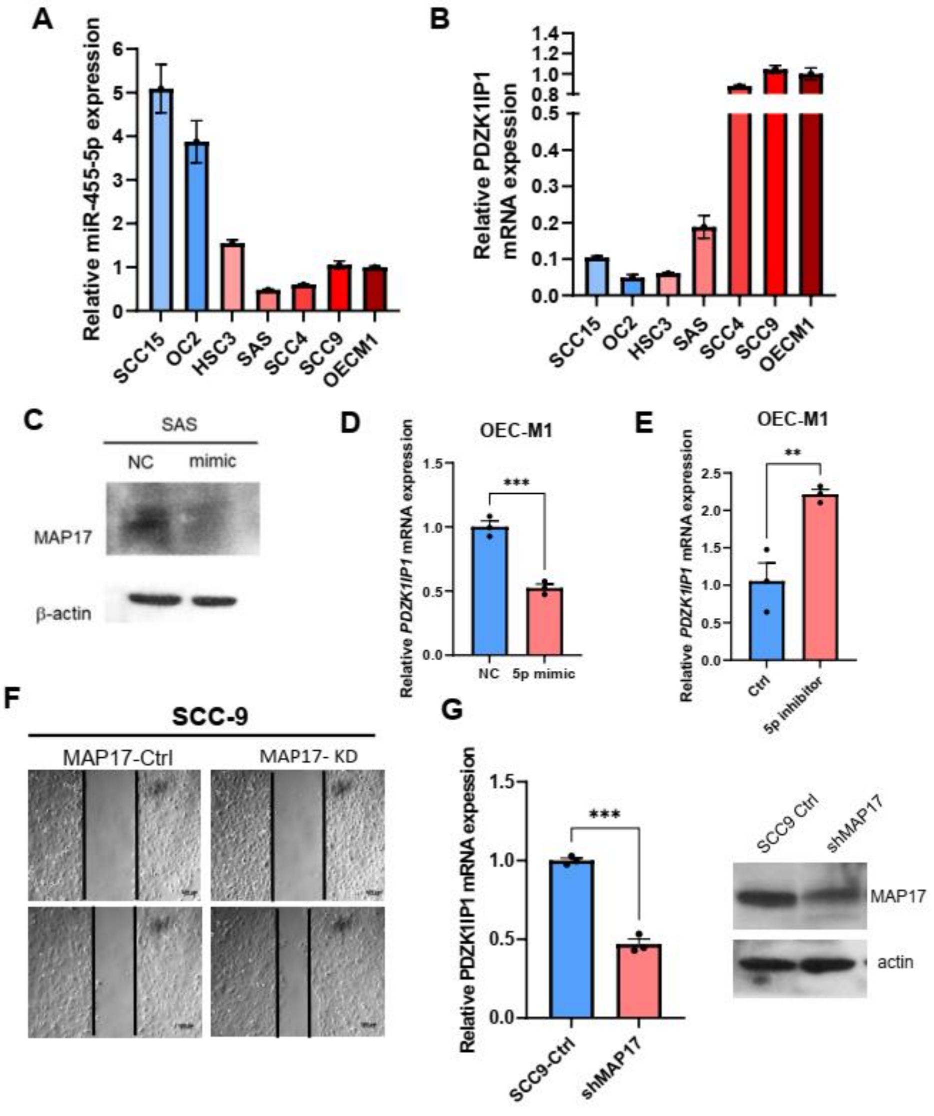

Figure S5. A-C Correlations among *PDZK1IP1* and *CDH1* (**A**), EMT score (**B**), and *LAMC2* (**C**) in dataset of NKCU-OrCA-40TN. **D** Recurrence-free survival of patients with high and low partial EMT scores in the NKCU-OrCA-40TN data set. **E-I** Correlations among miR-455 and *PDPN* (**E**), *LAMC2* (**F**), partial EMT score (**G**) or *PDZK1IP1* and SPRR1B (**H**), *S100A9* (**I**) in TCGA database. **J** Overall survival curves of patients with high or low partial EMT scores in the TCGA database. Correlation p value was determined through Pearson correlation analysis. Statistical p value of the survival curve was determined using a Mantel–Cox log-rank test.

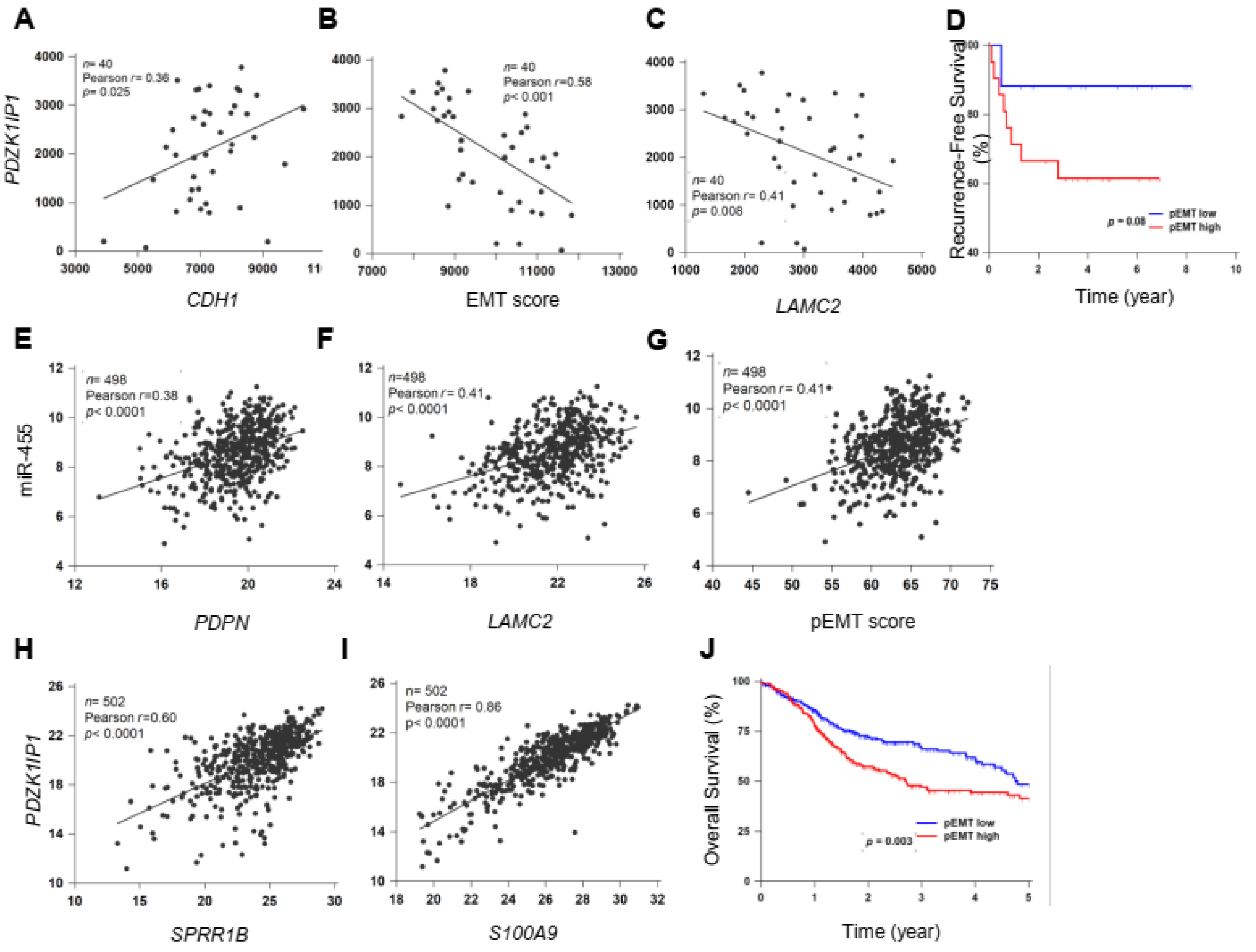

Figure S6. A Relative miR-455-5p expression levels in three different OEC-M1 cells. 5p OE: OEC-M1 pLe-455-5p cells; 5p OE+MAP17OE: OEC-M1 pLe-455-5p cells with *PDZK1IP1* overexpression plasmids. **B** Relative *PDZK1IP1* mRNA expression levels in three different OEC-M1 cells. **C** Cell migration ability was assessed by wound-healing assay. **D** Cell invasion ability was assessed by two-chamber transwell invasion assay **E** Relative mRNA expression levels of EMT-associated genes (*CDH1*, *VIM*, and *SNAI2*) and partial EMT-associated genes (*LAMB3*, *PDPN*, and *LAMC2*). **F** Kaplan–Meier survival curve of patients with miR-455-5p high (or low) and MAP17 low (or high). *$p < 0.05$, **$p < 0.01$, ***$p < 0.001$, ****$p < 0.0001$ by two-tailed Student's t test.

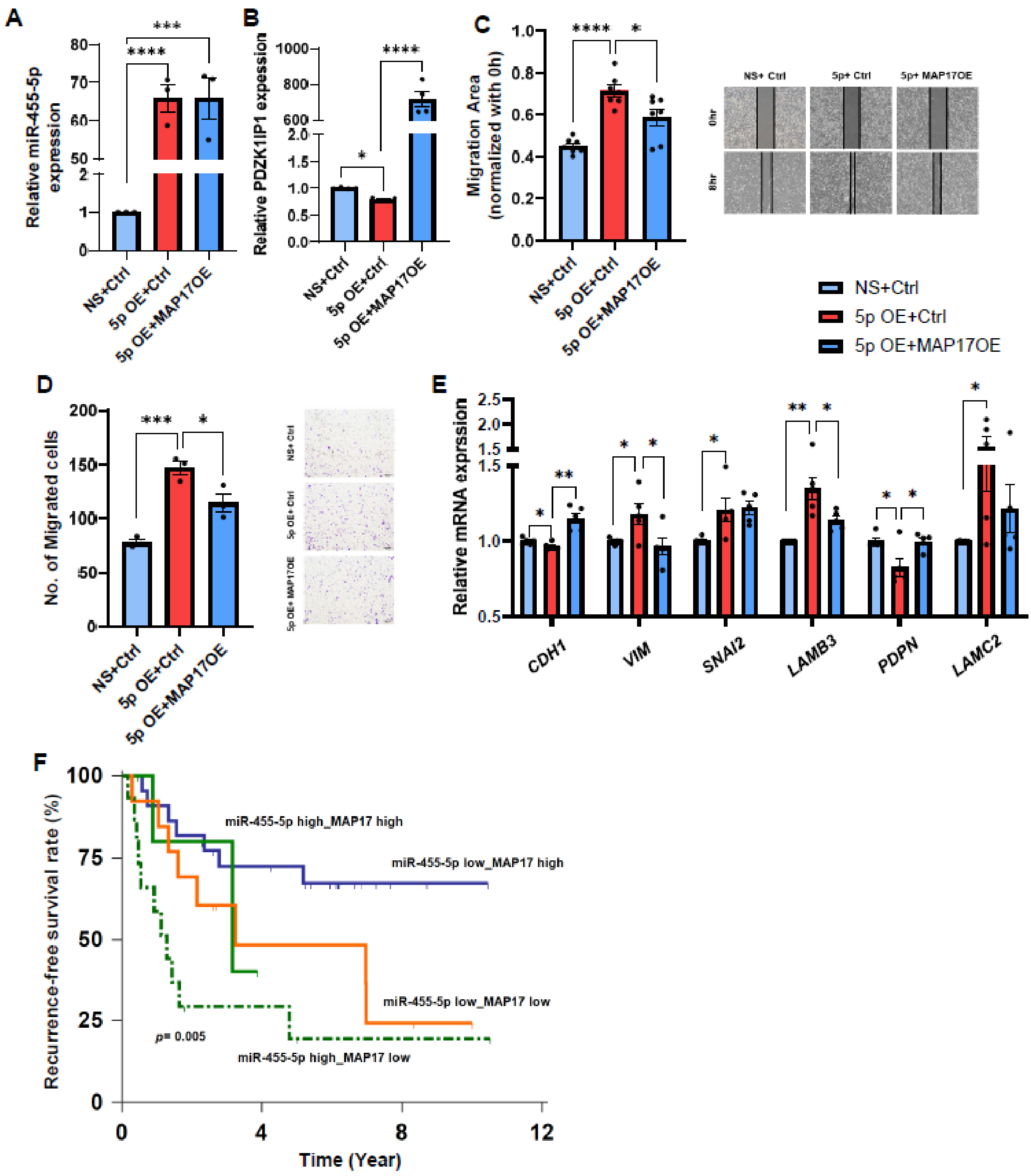

Video S1A. Time-lapse microscopy exhibited migration pattern of HSC-3 cells with vector control in 2.5D collagen-coated culture system.

Video S1B. Time-lapse microscopy exhibited migration pattern of HSC-3 cells with miR-455-5p overexpression in 2.5D collagen-coated culture system.

Video S2A. Time-lapse microscopy exhibited migration pattern of SAS cells with vector control in 2.5D collagen-coated culture system.

Video S2B. Time-lapse microscopy exhibited migration pattern of SAS cells with *PDZK1IP1* overexpression in 2.5D collagen-coated culture system.

Supplementary Table 1. MiR-455-5p and MAP17 expression in relation to clinical parameters and pathological characteristics of OSCC patients

Parameters	miR-455-5p* low (n=35)	miR-455-5p high (n=21)	*P* value	MAP17* low (n=28)	MAP17 High (n=28)	*P* value
T						
T1-T2	16	9		11	14	
T3-T4	19	12	0.8365	17	14	0.4242
N						
negative	24	8		12	20	
positive	11	13	**0.027**	16	8	**0.0323**
TNM stage						
I-II	12	4		5	11	
III-IV	23	17	0.2259	23	17	0.0786
PNI						
negative	18	10		12	16	
positive	17	11	0.7844	16	12	0.2894
LVI						
negative	21	9		11	19	
positive	14	12	0.2172	17	9	**0.0336**
ECE						
negative	8	9		13	4	
positive	3	4	0.8542	3	4	0.1201
Poor risk						
negative	16	5		7	14	
positive	19	16	0.1043	21	14	**0.0555**
MAP17						
Low	13	15				
High	22	6	**0.0138**			
Vimentin*						
Low	26	8		8	26	
High	9	13	**0.0078**	20	2	**<0.0001**

PNI: perineural invasion; LVI: lymphovascular invasion; ECE: extracapsular extension; Poor risk: patients with PNI, ALI, or ECE.

* Divided into high and low groups according to the mean expression values or mean H-scores.
